# The Effect on Mental Health of a Large Scale Psychosocial Intervention for Survivors of Mass Violence: A Quasi-Experimental Study in Rwanda

**DOI:** 10.1371/journal.pone.0021819

**Published:** 2011-08-08

**Authors:** Willem F. Scholte, Femke Verduin, Astrid M. Kamperman, Theoneste Rutayisire, Aeilko H. Zwinderman, Karien Stronks

**Affiliations:** 1 Department of Psychiatry, Academic Medical Center, University of Amsterdam, Amsterdam, The Netherlands; 2 Equator Foundation, Diemen, The Netherlands; 3 Department of Psychiatry, O3 Mental Health Care Research Center, Erasmus MC, Rotterdam, The Netherlands; 4 Episcopal Church of Rwanda, Diocese of Byumba, Byumba, Rwanda; 5 Department of Clinical Epidemiology, Biostatistics and Bioinformatics, Academic Medical Center, University of Amsterdam, Amsterdam, The Netherlands; 6 Department of Public Health, Academic Medical Center, University of Amsterdam, Amsterdam, The Netherlands; The University of Queensland, Australia

## Abstract

**Background:**

War has serious and prolonged mental health consequences. It is argued that post-emergency mental health interventions should not only focus on psychological factors but also address the social environment. No controlled trials of such interventions exist. We studied the effect on mental health of a large scale psychosocial intervention primarily aimed at social bonding in post-genocide Rwanda. The programme is implemented at population level without diagnostic criteria for participation. It is open to any person older than 15 years, and enables participation of over 1500 individuals per year. We postulated that the mental health of programme participants would improve significantly relative to non-participants.

**Methods and Findings:**

We used a prospective quasi-experimental study design with measurement points pre and post intervention and at 8 months follow-up. 100 adults from both sexes in the experimental condition entered the study; follow-up measurements were taken from 81. We selected a control group of 100 respondents with similar age, sex and symptom score distribution from a random community sample in the same region; of these, 73 completed the study. Mental health was assessed by use of the Self Reporting Questionnaire (SRQ-20), a twenty item instrument to detect common mental disorders in primary health care settings. Mean SRQ-20 scores decreased by 2.3 points in the experimental group and 0.8 in the control group (*p* = 0.033). Women in the experimental group scoring above cut-off at baseline improved with 4.8 points to below cut-off (*p*<0.001). Men scoring above cut-off at baseline showed a similar trend which was statistically non-significant. No adverse events were observed.

**Conclusions:**

A large scale psychosocial intervention primarily aimed at social bonding caused a lasting improvement of mental health in survivors of mass violence in Rwanda. This approach may have a similar positive effect in other post-conflict settings.

**Trial Registration:**

Nederlands Trial Register 1120

## Introduction

Violent conflict has serious and prolonged mental health consequences [1]–[6]. Post-emergency mental health interventions are mostly aimed at persons at risk of psychiatric disorder, particularly post-traumatic stress disorder (PTSD) [7]. However, opinions differ regarding the value of such psychological trauma-focused care [8]. As organized violence affects individuals as well as communities and social institutions, it is argued that mental health interventions should not only focus on internal psychological factors but also address aspects of the social environment which could promote healing and adaptation [9]. Unlike trauma-focused approaches, psychosocial interventions focus primarily on stressful environmental conditions such as the division within communities, the destruction of social networks and the resulting loss of social and material support. Altering these conditions may foster people's inherent capacity to recover [10], cause improvement in nonspecific symptoms among persons with and without specific disorders and, in some cases, be enough to reduce symptoms below the threshold of clinical disease [11]. Psychosocial interventions are preferably implemented at population level and directed at groups rather than individuals. Group interventions have shown to have a positive impact on health outcomes in other areas of public health [12], [13]. In war-affected societies a particular objective may be the restoration of social connectedness and mutual support. To date, the literature on humanitarian responses to disaster does not reflect any substantive discussion of comprehensive psychosocial interventions [14], and no controlled trials of such interventions exist. We carried out a controlled study to assess the effect on mental health of a psychosocial intervention programme which makes use of a therapeutic group approach called sociotherapy. It primarily aims at social bonding and secondarily at mental health improvement. We postulated that the mental health of programme participants as assessed with use of the Self Reporting Questionnaire (SRQ-20) would improve significantly relative to non-participants.

The intervention has been taking place since early 2006 in Gicumbi district (the former Byumba province) in the north of Rwanda, and is presently still running. The population of Rwanda experienced extreme violence during the genocide of 1994, when within a three month period about 800,000 people were killed; roughly two million refugees left the country, and around one million people were internally displaced. Only a few studies examined the mental health status of Rwanda's post-genocide population, but all show high rates of mental health disorders, particularly depression and PTSD [15]–[21].

The sociotherapy programme is community-based, that is, it is carried out by trained Rwandan community leaders, and is implemented at population level. It enables over 1500 beneficiaries per year to participate. No diagnostic criteria for participation have been defined, as the programme aims to be accessible to all community members.

This study took place from October 2007 to September 2008, preceded by a pilot study over 2005–2006. Measurements were taken pre and post intervention and at 8 months follow-up.

## Methods

The protocol for this trial and supporting CONSORT checklist are available as supporting information; see Checklist S1 and Protocol S1.

### Ethics statement

Approval for this study was gained from the Medical Ethics Committee of the Academic Medical Center in Amsterdam, Netherlands. This included approval for the consent procedure used (see under paragraph ‘Interviews’).

### Intervention

Sociotherapy has its roots in England during the second world war, when society had to cope with many psychiatric casualties [22]. The technique therapeutically uses interaction between individuals and their social environment to help subjects to re-assess and re-define values, norms, relations and possible collaborations. The principal premise is that reaching a certain level of mutual respect, trust and care in group interaction helps to increase the problem solving capacity and subjective mental health in individual group participants. In sociotherapy with survivors of systematic violence, safety and the setting of democratic rules are additional primary objectives. The intervention does not specifically aim at sharing or processing traumatic memories. Trauma symptoms are addressed through psycho-education and advice. Key elements of the working methods are debates and the exchange of experiences and coping strategies among participants, exercises, games and mutual practical support.

The sociotherapy programme studied here was set up in collaboration with the Église Episcopale au Rwanda (EER), funded by the development organization Cordaid and technically supported by a Dutch agency, Equator Foundation. Approval was given by regional and national authorities in Rwanda. Wide support on community level was gained through public acclamation by the EER. Close collaboration with local staff, allowing local control and embracing local social manners and values were key to the programme's viability. The programme was open to any adult (≥16 years) wanting to participate. Given the large number of applications over the course of time it appeared to fulfill a widely felt need. Also, community members could personally be invited when considered psychosocial problem cases by sociotherapy group leaders. Groups contained 10 to 15 participants and were mostly mixed: both sexes, various ethnic backgrounds, wide age distribution. Forty-five groups ran simultaneously, having weekly meetings over a period of 15 weeks, lasting 3 hours each. Participants were extremely compliant, although there was no material gain by attending. Group leaders were local people, familiar with the region's history and current living situation; they had received 3 months of training from Equator staff and were regularly supervised. They received no fees, though travel expenses were reimbursed.

Sociotherapy's most prominent principles and phases have been described elsewhere [22], [23]. The method is not strictly protocolized. In non-clinical, international settings it is essential to continuously tailor it to the actual context and group. Group leaders are allowed to attune their routines to the characteristics of their groups (e.g., degree of trust, nature of problems) and to their own affinity and experience, putting different emphases on elements like rules, role plays, and spirituality. For example, group leaders who are pastors may stimulate praying and singing, while teachers may encourage role plays and debate about social rules; others again may take a less active role, supporting the group to share experiences. There were some core principles, however, that all group leaders complied to: two-way communication, shared leadership, consensus in decision-making, and social learning through actual social interaction. Additionally, each subsequent phase of a group had a different focus, notably safety, trust, care, respect, rules and memories. While the exact working mechanism of sociotherapy is not known, it is plausible that in Rwanda it brings people whose relationships have been severely ruptured closer to one another [24].

### Instrument

Data was collected at the start of the intervention (baseline, T0), directly after (T1), and at 8 months follow-up (T2). Demographic data (sex, age, level of education and socio-economic status) were documented. Assessments were done by use of the Self Reporting Questionnaire (SRQ-20), an instrument developed by the World Health Organization (WHO) for screening for common mental disorders in primary health care settings. The instrument is often used in developing countries [25], [26]. When patients are literate it can be self-administered, but in developing countries it is usually administered by lay interviewers. It consists of 20 yes/no questions about mood, thinking capacity, feelings of anxiety and physical well-being. ‘Yes’ answers result in a higher score, meaning a poorer mental health condition. Cut-off points vary considerably depending on setting and culture. A cut-off point of 7/8 is widely used [27].

We (back-)translated the SRQ-20 to the local language, Kinyarwanda, and validated it for the actual context. The capacity of the SRQ-20 to identify probable psychopathology proved to be sufficient for men (AUC = 0.74) and women (AUC = 0.76). Reliability was considered to be good (Cronbach's α = 0.83). The optimal cut-off point was 7/8 for men and 9/10 for women (manuscript under review). We also validated the SRQ-20 for its capacity to assess change in symptom severity over time. The instruments factor structure proved to be time invariant; the number of factors, factor loadings and covariances of factors remained equal over time.*

### Participants

A pre to post intervention test performed as a pilot study among sociotherapy participants (n = 77) showed a decrease of 2.7 (sd 4.2) of SRQ-20 mean scores (effect size 0.6). To establish a 2.7 effect with a standard .80 power, a minimum of 30 respondents in both the experimental and control group would be needed. To be on the safe side we aimed at larger numbers (n = 100) per study group. We did not aim at even higher numbers because of limited time, the large distances between the areas where respondents resided, and the low drop out rate during our pilot study.

*Experimental group.* Out of 45 sociotherapy groups starting simultaneously, the sociotherapy programme staff selected 10 groups through connivance sampling, balancing the gender ratio. These appeared to be large groups, and an unexpected high number of 133 participants showed up at the interview sites. At T0 we interviewed all 133, but at T1 and T2 we had to restrict ourselves due to limited time and human resources. Therefore, we invited a random selection of 100 out of the 133 to form our experimental group.

*Control group.* We applied the following procedure to compose a control group that was equivalent at baseline with regard to our main outcome measure, the SRQ-20 score. During our pilot study, 2.5 times more respondents in the experimental group (n = 97) had baseline scores above cut-off than in the control group (n = 229). For the actual study we therefore aimed to interview 2.5 times (n = 250) more respondents than in the experimental group, to later select 100 out of these to compose a control group. We identified five regions within Gicumbi district where the programme was not or had not been running so far, or for practical reasons would not start over the upcoming year. It could be assumed the inhabitants of these regions had experienced similar trauma exposure. Here, we randomly selected respondents through convenience sampling. Interviewers started at the top of a hill or in the centre of a village and each walked down a different footpath towards scattered houses or huts. An equal number of men and women, at home or in the fields, were randomly chosen and asked to participate. Finally 251 respondents were interviewed. After analysis of the data collected, we selected a group of 100 out of these for which the distribution of SRQ-20 scores matched that of the intervention group. For this purpose we used 8 clusters of scores (0–1, 2–3, 4–5, 6–7, 8–9, 10–12, 13–15, 16–20) and from each cluster randomly selected a number of respondents equal to the corresponding cluster in the experimental group. This final selection of 100 constituted our definite control group.

### Interviews

Eight local interviewers were recruited; all were sociology students at the ‘Institut Polytechnique de Byumba’ in Gicumbi. Their one-week training addressed the principles of a longitudinal study design, interviewing techniques and our measuring instrument. They were involved in making the wording of the questions acceptable and understandable for people in Gicumbi [28]. Informed consent was obtained by use of an explanatory text, which because of the high illiteracy rate was read aloud. In case of refusal, demographic data and reasons for refusal would be requested and documented, but no-one refused.

For determination of the socio-economic status (SES) our interviewers approached respondents of the control group at, or near, their homes, and scored the SES by judging the state of the houses. Participants of the experimental group, however, were interviewed at the spot of their meetings, and were asked to describe the state of their houses themselves.

### Statistical analysis

The repeated measures of our primary outcome, the total SRQ-20 score, were analyzed with a linear mixed-effects model (SPSS 16.02) using intervention (participants versus controls), time (T0, T1, T2) and their interactions as fixed-factors. Sociotherapy groups and control group areas, and respondents within the sociotherapy groups and the control group areas were random factors. An intention to treat analysis was employed, in which all available measurements of all respondents were analyzed according to the mixed-effects model. Missing data were considered to be missing at random in the repeated measures model. No assumptions on the covariances between the repeated measures were made (covariance type: unstructured). The primary hypothesis on the effectiveness of the intervention was tested with the p-value of the interaction test between time and intervention; a *p*-value of 0.05 or less was interpreted as statistically significant. We analysed for the sample as a whole, and stratified for sex and separate sociotherapy groups. Total SRQ-20 scores are presented with the mean. Significant interaction effects are presented with estimates of the differences in mean SRQ-20 scores relative to baseline with 95% confidence intervals. We also present the reliable change index according to Jacobson and Truax [29].

In addition, we extended the same repeated measures model with the baseline SRQ-20 score as a variant, and then analyzed SRQ-20 score changes. Since the results were comparable, we do not report these. To quantify the variability of the score change between the different sociotherapy groups we calculated the within and between sociotherapy group variances of the score change between T0 and T2 per participant as well as the average score change per sociotherapy group with its 95% confidence interval; here, we calculated the expectation of the posterior distribution of random effects with an empirical Bayesian analysis.

## Results

### Baseline characteristics

**Figure 1:**
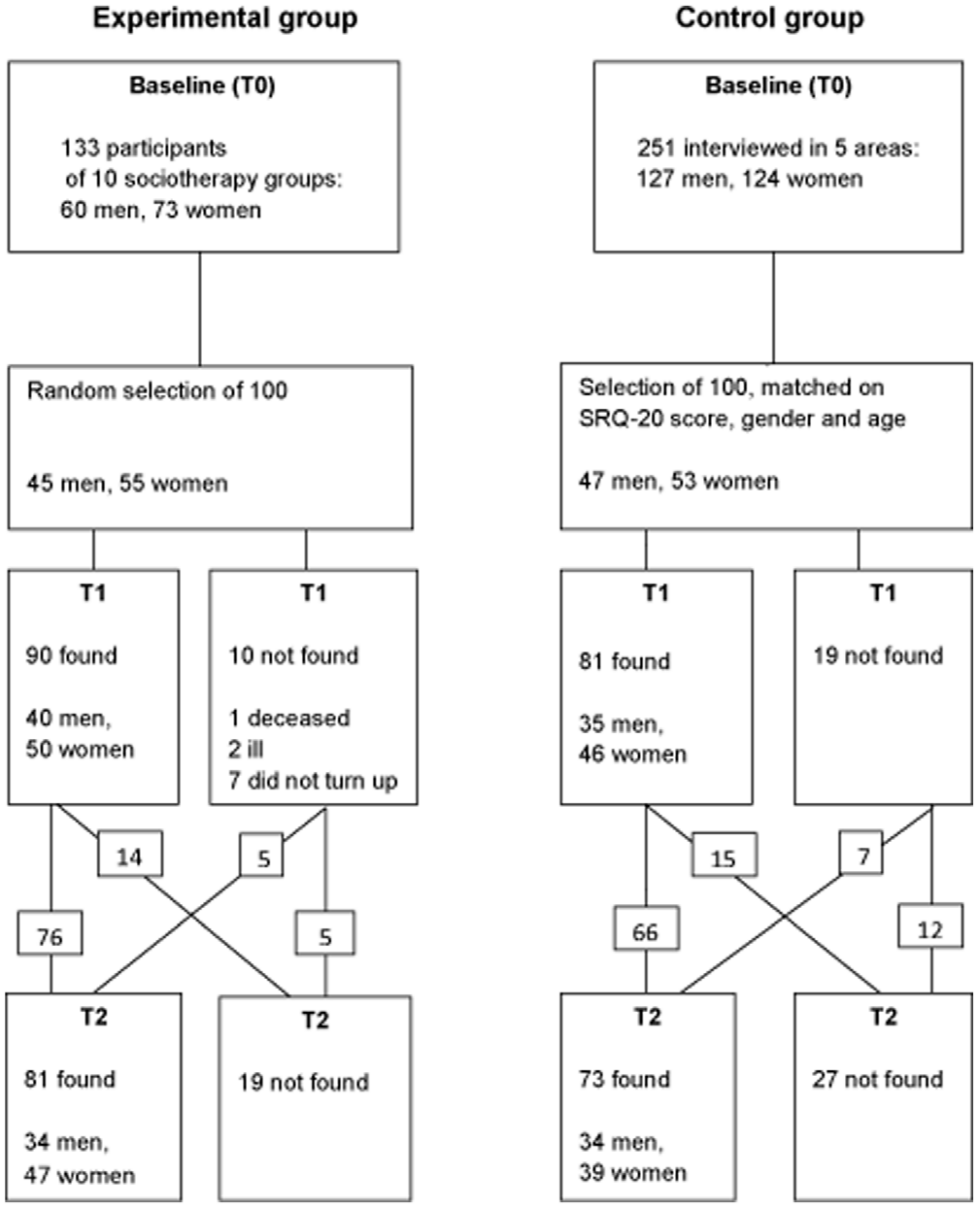
Flow chart of the composition of the study population at three measurements.

Baseline measurements took place in September 2007. The two study groups matched on SRQ-20 score distribution, sex and age at baseline (see Table 1). At T1, in January 2008, 90 subjects from the experimental group and 81 from the control group were interviewed, and at T2 (eight months later) 81 and 73, respectively. Of these, only 76 and 66 had been interviewed at both T0 and T1 (see Figure 1). The study groups showed no significant difference in level of education. They differed slightly in SES, with an overrepresentation of both lowest and highest SES groups in the control group.

**Table 1:** Socio-demographic characteristics of experimental and control group at baseline.

	Experimental group (n = 100)	Control group (n = 100)	
**Sex**			
Male	45 (45%)	47 (47%)	
Female	55 (55%)	53 (53%)	
P-value (Chi^2^)			0.78
**Mean Age**			
years	34.9	38.5	
min-max	16–76	16–73	
standard deviation	15.8	14.1	
P-value (T-test)			0.10
**Education**			
nil	48 (48%)	54 (54%)	
primary	42 (42%)	34 (34%)	
secondary 1–3	9 (9%)	9 (9%)	
secondary 4–7	1 (1%)	3 (3%)	
P-value (Chi^2^)			0.53
**SES**			
marginal	6 (6%)	13 (13%)	
poor	83 (83%)	66 (66%)	
sufficient	11 (11%)	21 (21%)	
P-value (Chi^2^)			0.022
**SRQ-20 score**			
mean	8.41	8.26	
standard deviation	5.05	4.83	
P-value (T-test)			0.83

### Drop out

Drop out was unexpectedly higher than during the pilot study (see Figure 1). Drop out from the experimental group was mainly caused by illness, leaving the programme for unknown reasons and communication problems about day and time of interviewing. One particular sociotherapy group contained scholars; at T2 they had finished school and had moved to different areas. Drop out from the control group was also caused by illness and communication problems, but mostly by moving house. Drop out did not differ significantly between the experimental and the control group (*p* = 0.79). Drop-outs at T1 or T2 from either study group did not differ significantly in sex, age or level of education. Neither was there a difference in sex, age, level of education and SRQ-20 scores between actual respondents and drop-outs at T1 and T2.

### Changes in SRQ-20 scores

**Figure 2:**
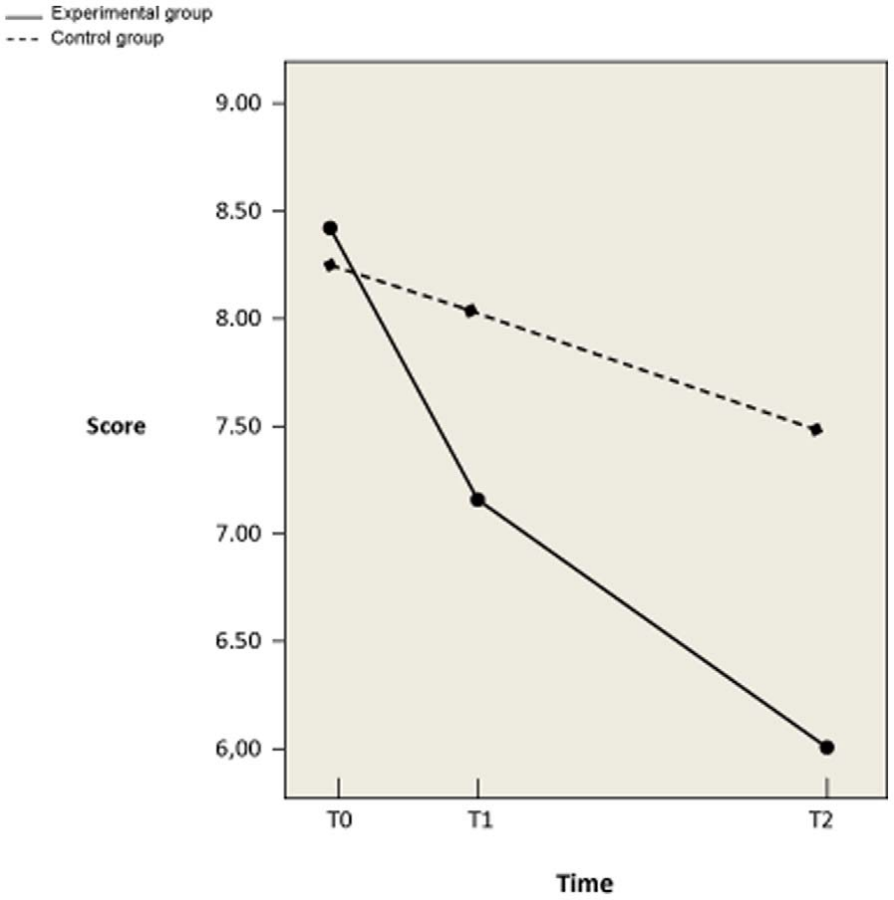
SRQ-20 score changes between T0 and T2 in experimental and control group.

Linear mixed-effects model analysis yielded a significant difference between the two study groups in decrease in mean SRQ-20 scores at follow-up (see Table 2 and Figure 2). From baseline to T2 the mean decrease was 2.3 in the experimental group versus 0.8 in the control group, meaning a difference in decreases of −1.59 (95% CI: −2.81 to −.38). The reliable change index was 0.61 for the experimental group and 0.20 for the control group. After stratifying for men and women, we noted a disparity in the time-intervention interaction. For women, there was a significant difference between the experimental and the control group. The estimate of the difference in decreases of SRQ-20 scores was −2.47 (95% CI: −4.14 to −.79). Men started with lower SRQ-20 scores in both groups; the groups did not differ significantly in the time-intervention interaction.

**Table 2:** Mean SRQ-20 scores, standard deviations, effect sizes (T0–T2) and *P*-values for experimental and control group.

	Total (n = 200)					Men (n = 92: exp 45, contr 47)					Women (n = 108: exp 55, contr 53)				
	T_0_	T_1_	T_2_	Cohen's d	*p*	T_0_	T_1_	T_2_	Cohen's d	*p*	T_0_	T_1_	T_2_	Cohen's d	*p*
**Mean (sd)**															
Experimental group	8.4 (5.0)	7.2 (4.6)	6.1 (3.9)	0.51		6.6 (4.6)	5.8 (4.8)	5.3 (4.0)	0.30		9.9 (4.9)	8.3 (4.3)	6.8 (3.8)	0.70	
Control group	8.3 (4.8)	8.1 (5.7)	7.5 (4.8)	0.17		6.5 (4.0)	5.8 (5.0)	5.6 (4.0)	0.22		9.8 (5.0)	10.0 (5.8)	9.2 (5.2)	0.18	
					0.033					0.852					0.011

We also focused on possible cases, that is: the 63% of females and 37% of males scoring above the respective cut-off values of 9 and 7 at baseline. Table 3 shows their numbers at each measurement.

**Table 3:** Numbers of possible cases in experimental and control group at each measurement.

	Men			Women		
	T0	T1	T2	T0	T1	T2
**Experimental group**	16	11	7	34	17	8
**Control group**	18	9	9	34	23	17

We then assessed the time-intervention interaction for these possible cases (see Table 4). The mean score of females in the experimental group dropped below cut-off at T1 and improved further at T2. The decrease is significantly larger than in the control group. The estimate of the difference in decreases was −3.08 (95% CI: −4.89 to −1.27). The mean scores of men also decreased in both study groups but these trends did not differ significantly, and neither subgroup reached a level below cut-off. Individual scores in women decreased to below cut-off in 19 out of 34 (56%) in the experimental group versus 7 out of 34 (21%) in the control group. In men, this was 7 out of 16 (44%) versus 5 out of 18 (28%).

**Table 4:** Mean SRQ-20 scores, standard deviations, effect sizes (T0–T2) and *P*-values for possible cases.

	Men (n = 34: exp 16, contr 18)					Women (n = 68: exp 34, contr 34)				
	T_0_	T_1_	T_2_	Cohen's d	*p*	T_0_	T_1_	T_2_	Cohen's d	*p*
**Mean (sd)**										
Experimental group	11.6 (2.9)	10.4 (3.9)	8.5 (4.1)	0.87		13.2 (2.4)	9.5 (4.3)	8.4 (3.5)	1.60	
Control group	10.7 (2.2)	8.5 (5.5)	8.0 (3.6)	0.90		13.1 (2.0)	12.6 (3.9)	11.4 (3.2)	0.64	
					0.621					<0.001

**Figure 3:**
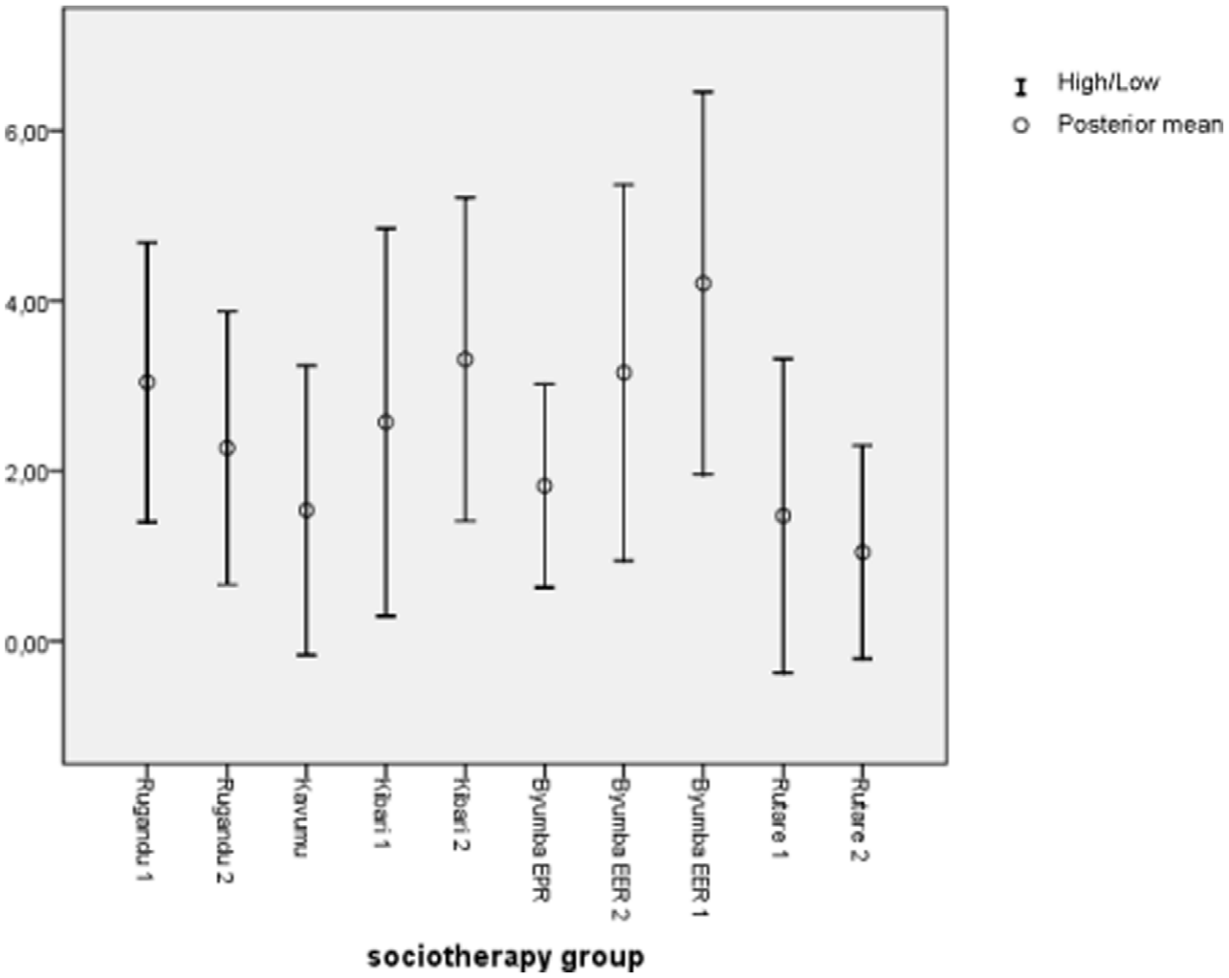
Expected mean SRQ-20 score changes between T0 en T2 of ten sociotherapy groups.

We then assessed the extent to which the sociotherapy effectiveness differed between groups. The variance between participants in the same group (the within groups variance) in SRQ-20 score change between T0 and T2 was 12.79. The between groups variance was 1.84, and therefore the intraclass correlation of the score change was 0.14, suggesting that about 14% of the total variability in the score change might be attributed to factors associated with specific sociotherapy groups. The posterior mean SRQ-20 score change for the ten sociotherapy groups is illustrated in Figure 3.

## Discussion

Our results suggest that the mental health of all survivors of mass violence studied here improved over time. Those who participated in the sociotherapy programme, however, showed an increased improvement over the duration of the intervention.This improvement continued after the intervention, and the difference in scores between the experimental and the control group was even larger at follow-up. This effect is significant in women, and seems to have clinical relevance: the mean SRQ-20 score of female possible cases in the experimental group dropped significantly, ending below cut-off. This corresponds with our finding that the individual scores of 56% of this subsample dropped to below cut-off, a substantially larger proportion than of female possible cases in the control group (21%). A significant improvement was not noted in male possible cases. However, improvement to below cut-off in male possible cases was more frequent in participants in the intervention programme than in the control group: 44% versus 28%.

As no quantitative outcome data of comprehensive psychosocial programmes in post-conflict settings exist so far, we relied on data from our pilot study to establish an appropriate study sample size. Our trial's methodological strengths include adequate follow-up rates, and use of a measure that was locally validated for use as a screening instrument as well as for measuring symptom change over time. We used a quasi-experimental design, composing a control group equivalent to the experimental group with regard to our main outcome measure and to sex and age. Although the latter group could be considered as help seeking while the first is a community sample, the demand for the programme has shown to be widespread from the start, and its existence was not yet known to control group respondents. Besides, we think that the ‘one-time opportunity’ character of the intervention starting at a certain location was a key determining factor for participation, rather than a worse-than-usual mental state or greater openness in candidate participants at the start. Additionally, given the similarity of the living conditions of both study groups, the risk of confounding bias may be considered minimal. Yet, as this is not a randomized trial, one cannot completely rule out the existence of hidden systematic group differences. A difference between the study groups was noted in SES at baseline. We do not think that this seriously impacted the actual equivalence of both groups. Gicumbi's population is extremely poor in general, and actually there is little real variety in SES. Possibly, the difference is caused by the method of SES determination. Contrary to the control group, participants of the experimental group described the state of their houses themselves. This may have resulted in a less divergent SES score distribution in the experimental group. A limitation of this study is that interviewers were not blind to the treatment condition, which may have affected the results. They were, however, in no way linked to the sociotherapy programme. Another limitation concerns the lack of detailed data on the proceedings of separate sociotherapy groups.

The applicability of the intervention may have been facilitated by its community-based and contextual sensitive nature, by the local prestige of its coordinators (EER), and by Rwanda's long history of organizing communities in group structures. The programme's impact may have been constrained, however, by the country's still paranoid atmosphere and the prevailing tendency of its inhabitants to keep problems inside, especially in men. Qualitative information consistently pointed out that men in Rwanda generally do not share emotional problems. This may have impacted data from male respondents and the way they actually participated in the intervention. Additionally, the lack of significant effect in men may be explained by better mental health at the start.

Trials on mental health interventions in post-conflict contexts are rare. The few interventions studied vary from those carried out by multi-disciplinary teams and targeting all help-seeking clients or only clinically indicated clients [30], [31], to interventions directed at psychiatric cases [32]–[34], or school-based programmes focusing on children [35]. To our knowledge no controlled trials exist of large scale, population level, psychosocial interventions for survivors of mass violence. Such interventions are in line with the IASC Guidelines on Mental Health and Psychosocial Support in Emergency Settings, a consensus document endorsed by all relevant players [36]. This is the first controlled trial of a psychosocial intervention of this kind. The intervention is community-based in the sense that it is owned and carried out by members of the local population. Its sustainability is shown by its ongoing implementation for over 4.5 years now, with over 7.000 participants so far. Our study findings indicate that such an intervention may be clinically relevant and beneficial to mental health problem cases, and that the programme as well as this study deserve replication in other post-conflict contexts. Future studies may establish if the difference in effect between the two sexes found here is related to the actual context or to the intervention method. By collecting data on methods used per group, future studies may also seek to identify favourable and adverse factors within the intervention's working methods.

## Supporting Information

Checklist S1 CONSORT Checklist.
(DOC)

Protocol S1 Trial Protocol.
(PDF)
