# Supplementary material for: The Effect on Mental Health of a Large Scale Psychosocial Intervention for Survivors of Mass Violence: A Quasi-Experimental Study in Rwanda
Source: PLoS One. 2011 Aug 8;6(8):e21819. doi: 10.1371/journal.pone.0021819 (PMC3152564; doi:10.1371/journal.pone.0021819)
Supplement: Protocol S1 — Trial Protocol. (PDF) [file pone.0021819.s002.pdf]

Project title:
Mental health, social functioning and social capital in Rwanda

Project leader:
WF Scholte MD, Academic Medical Center, Dept of Psychiatry
Academisch Psychiatrisch Centrum, PA-1.126, Meibergdreef 5, 1105 AZ Amsterdam
Tel.: 020. 8913571. Email: w.f.scholte@amc.uva.nl

Summary

Non-western, traumatized survivors of war or political violence frequently display complex trauma-related psychopathology, with anxiety, depressive and cognitive disturbances exceeding the domain of posttraumatic stress disorder. Besides this, most patients suffer from feelings like shame, guilt, distrust and alienation. Such manifestations complicate social functioning and interpersonal contacts in communities where social structures and cohesion already have been damaged by human violence.
This study concerns a therapeutic group approach carried out in post-genocide Rwanda. Using a trauma perspective, the intervention method is specifically tailored to survivors of systematic violence. Next to individual recovery it aims at the restoration of safety, mutual respect, trust, care, and the setting of democratic rules.
By use of a prospective controlled concurrent groups design, the study measures the intervention's effects on mental health, social functioning and social connectedness. Additionally, the study will seek to obtain data about changes in alcohol abuse and partner violence, as these are frequent manifestations of posttraumatic psychopathology.
Social connectedness is operationalized through the concept of social capital. There is growing international interest in the notion of social capital, as reserach data suggest it is connected to health and well-being. The concept and its measurement need further development, though, and this study contributes to this goal.
In the study as a whole, the methods used are exemplary for those used in international mental health research. Amongst those are the complementary use of qualitative and quantitative research methods, the local validation of instruments and the development of instruments specifically meant for use in the local context. In light of differences in cultural backgrounds and semantics, these methods are relevant for studies concerning migrants in our country as well.
Findings about the relation between mental health and social connectedness will impact future intervention planning in international humanitarian and development aid, and they are also relevant for mental health care as provided to refugees and to migrants in general in our country.

Problem definition

The Equator treatment program at the Academic Medical Center in Amsterdam focuses on refugees from war and political violence suffering from severe trauma-related psychopathology. Over the past 3 years patients originating from ± 30 different countries were treated. The treatment method aims to simultaneously promote mental health and social connectedness/integration. Equator also carries out aid programs in post-conflict areas overseas.
There is great variety in the historical, political, social and cultural contexts of wars and periods of political repression, and the mental health needs of survivors cannot be addressed adequately without tailoring interventions to these contexts. The help provided by different aid organizations under the heading of 'psychosocial' or 'mental' is diverse, although most programs focus on individual counseling. Such interventions have become the subject of considerable debate surrounding their adequacy and effectiveness (ref.1,2). Up to now, only few studies have scientifically examined mental health interventions in developing countries, and even less in areas of systematic violence. Among the rare examples of randomised controlled trials are the studies of Bolton et al (ref.3) who compared the effects of group interpersonal therapy with creative play in adolescent survivors of war and displacement in northern Uganda; Bass et al (ref.4) who compared group interpersonal psychotherapy with treatment as

usual in depressed rural Ugandans; and Neuner at al (ref.5) who compared narrative exposure therapy, supportive counseling, and psychoeducation for treating posttraumatic stress disorder (PTSD) in a settlement for Sudanese refugees.
Compared to western patients, non-western survivors of systematic violence display a broader spectrum of trauma-related psychopathology (ref.6,7). Affective (mostly anxiety and depression) and cognitive disorders, among which serious psychotic symptoms, frequently exceed the domain of PTSD and dominate the clinical picture. The adaptive systems, supportive to the individual's psychosocial equilibrium, that are most at risk in case of systematic violence, are: personal safety, attachment and bond maintenance, identity and role functioning, justice, and existential meaning (ref.8). Consequently, apart from showing psychiatric symptoms as mentioned above, severely affected patients also suffer from a loss of core beliefs and values, a sense of being permanently damaged, feelings of shame and guilt, distrust and alienation from others. The overall psychosocial consequences of war and political violence are far more than the sum of individual psychological symptoms. Systematic violence erodes a community's fabric and its cultural values, and individual recovery seems to be impacted by a community's ability to re-find its social structure (ref.9). A therapeutic method which uses a trauma perspective and simultaneously focuses on social re-bonding seems to be suitable to post-conflict settings. The intervention method studied through this project adapts to these conditions. It involves a group approach tailored to survivors of systematic violence, aiming at the restoration of safety, mutual respect, trust, care, and the setting of democratic rules.
The country of Rwanda experienced extreme violence during a genocidal three months period starting April 1994. Some 800.000 people were killed, roughly 2 million refugees left the country, and around 1 million people were internally displaced. Many Rwandans are still haunted by the genocide's terror. Only few studies exist to provide mental health morbidity estimates in post-genocide Rwanda. Bolton et al (ref.10) found that 15.5% of a random community sample of 368 adults met criteria for current major depression. Schaal & Elbert (ref.11) interviewed 68 orphans; 44% had PTSD. Pham et al (ref.12) found that 24.8% of 2091 randomly selected adults met symptom criteria for PTSD. In 100 widows Hagengimana et al (ref.13) found 35% suffered a panic disorder. Other studies either did not use diagnostic criteria or used a non-specific screening tool (ref.14,15).
At the request of, and in collaboration with, the Église Episcopale au Rwanda (EER), and financially funded by the development organization Cordaid, Equator started an intervention program in the Rwandan province Byumba in September 2005. The program, which will run for 6 years, will enable ± 1500 beneficiaries per year to participate in sociotherapy groups under the lead of trained local staff. The composition of these groups is mixed (both sexes, various ethnic backgrounds, wide age distribution). For reasons mentioned under 'Strategy', no strict criteria for participation have been defined. The intervention method is innovative because of its double focus on mental health recovery and social connectedness, as well as through its applicability to multiple settings; no earlier studies exist to provide an evidence base. This project concerns a controlled effect study of the aid program in Rwanda.

Relevance

This project will add to the evidence about the treatment of complex trauma-related psychopathology in non-western survivors of systematic human violence. Up to now, most treatment studies concerning psychological trauma relate to PTSD and/or depression and mainly focus on mental health. This project is innovative because it studies the effects of an intervention method that applies to more complex symptomatology and that focuses on social aspects as much as on individual pathology. The study is also relevant given the great lack of scientific evidence founding humanitarian and developmental aid programs worldwide. The method studied, however, serves the treatment of refugees in our country as well. Actually the effect study is relevant to all Dutch mental health care for migrants, because of the intervention's double focus on mental health and social connectedness/integration.
In our country there is little systematic academic attention for international mental health. The latter concept applies to migrants to the Netherlands as well as to populations worldwide. Core elements of this discipline are: intercultural communication, cross-cultural clinical diagnostics and treatment, worldwide epidemiology of psychiatric illness, overseas indigenous nosologies and explanatory models, public mental health policies, and the integration of mental health care at the primary care level. In

cross-cultural research, the parallel use of qualitative and quantitative methods and the cultural and language-specific validation of research instruments play an important role. This project will bring forth a specialist with clinical and scientific competencies specific to this field, which has great relevance in our continuously globalizing and multiculturalizing society.
The project will be carried out in close collaboration with the division of Culture, Health and Illness of the LUMC in Leiden (Prof. dr JM Richters). This partner conducts and supervises qualitative interviews and analyses to contribute to the local validity of research instruments, and later to help interpret quantitative study outcomes. Such cross-fertilization is not widely used in our country. Future publications of the study will add to the familiarity with this working method and to the acknowledgement of its value among researchers.
In this study social connectedness/integration is operationalized through the concept of social capital (ref.16,17). The notion of social capital refers to the existence of active ties between individuals, leading to networks wherein social cohesion and cooperation take place. The relevance of social capital is recognized by the World Bank, which invests in projects helping to define the concept and measure it. Social capital seems to be connected to economic growth as well as to health and well-being. Yet, the complete picture of the relation between social capital and health is still confusing, and there is a need to further develop the theory and definition of the notion and its measuring instruments. In the present study one of the better researched instruments to record the presence of social capital, the Social Capital Assessment Tool, short version (ref.18), is adapted and validated locally. The pilot of the study already yielded surprising data with regard to the instrument, with relevance within the whole of the objectives of the World Bank as mentioned above. Additionally, the study will increase our understanding of the relation between social capital and mental health, which is unknown as yet.
Parallel to this study, a controlled effect study of the treatment program in Amsterdam is carried out, financed by the European Refugee Fund. As the latter study examines the same double treatment focus, it will allow for even stronger application of findings of the Rwanda study to migrants in our country.
Populations living in post-conflict areas generally report high prevalences of mental health symptoms, but studies seldom address the functional impairment attributable to these symptoms (ref.9). It is debated if such findings either represent actual psychopathology or reflect a normal response to severely abnormal circumstances (ref.19). This study seeks to also map out social functioning, which is relevant to establish if symptoms reported through a structured interview indeed relate to impairment, and therefore can be considered to reflect psychopathology.
The intervention studied is considered as highly relevant in its recipient country. During a recent national conference in Rwanda’s capital Kigali, the Rwandan Ministry of Health recommended the method to be implemented nation-wide, and a delegation from DR Congo asked for implementation in that country. By now both extensions are in preparation.

Objective

The study aims to establish the effects of a therapeutic group intervention called sociotherapy, which is specifically tailored to traumatized survivors of systematic violence (war, political violence) displaying a broad spectrum of affective and cognitive disturbances. It is hypothesized that mental health symptoms, social functioning and social capital (a concept reflecting social connectedness) are positively impacted by the intervention, and that all three are interrrelated. It is also assumed that the intervention has a positive influence on two specific manifestations of posttraumatic psychopathology: alcohol abuse and partner violence.
This study examines the intervention as implemented in post-genocide Rwanda, Byumba province. Additionally, the study aims to establish the local validity of instruments to measure mental health, social functioning and social capital.

The research questions are:

1) Does sociotherapy impact mental health?
2) Does sociotherapy impact social functioning?
3) Does sociotherapy impact social capital?
4) Are mental health, social functioning and social capital interrelated?

5) Can local validity of three instruments (SRQ-20, BSFQ and SA-SCAT) be established to measure mental health, social functioning and social capital, respectively?
6) Does sociotherapy impact alcohol abuse?
7) Does sociotherapy impact partner violence?

Strategy

1. INTRODUCTION

1.1 Location

As the study population of this project lives in Rwanda, qualitative study elements, training of local research staff and structured interviewing will take place in that country, over separate time periods of several weeks each. During these periods the study's junior researcher, a resident in psychiatry, will be supervised by the project leader who will be on the spot as well at the time. Data collected will be coded to ensure anonimity, entered locally, and taken to Amsterdam. Filled out questionnaires will be stored and locked locally. Respondents' personal details will be stored and locked separately, in another building.
In between working periods in Rwanda the study continues in Amsterdam. The junior researcher will then be responsible to search and review all relevant literature; analyse data collected; discuss outcomes with the Rwandan counterpart and the other project members; and prepare to report study findings through presentations and publications. During the study's first year the junior researcher will be located at the Academic Medical Center (AMC), just as the project leader and the project's statistical expert who both will provide ongoing supervision and support, and the promotor. She will be offered AMC training courses in various elements of clinical research and statistics, and attend in-house and external scientific meetings and conferences.

1.2 Intervention, adaptations and consequences for research

The group intervention to be studied was developed for application in a clinical setting in Amsterdam, but now is applied at community level in a low-income country. In both settings similar principles underlie the intervention, and similar objectives are aimed at. Simultaneous to this study, research is carried out to measure the effects of the intervention in Amsterdam on (amongst others) the same variables: mental health, social functioning and social capital. In the Amsterdam study (Current Controlled Trials #: ISRCTN52861559), the experimental group is formed by clients of a treatment program for traumatized refugees; the control group is a population sample of refugees living in the Amsterdam region, matched on sex, age, ethnicity and living area.
Introduction of the intervention in a Rwandan post-Genocide community which is characterized by great sensitivity, not only needed the explicit approval of the program by regional and national authorities. Also, successful implementation was unthinkable without wide support on community level, which was gained through public acclamation of the program by its coordinators' influential local counterpart (EER). Additionally, in a setting not familiar to its Dutch coordinators, a bottom-up approach and close collaboration with local staff, embracing current local social manners and values, were crucial elements at all stages of the program implementation. Consequently, the actual set-up of the intervention was determined by the basic principles of the method as much as by local factors. The latter is most obviously reflected by the control over participation criteria by the community members themselves, who considered the intervention's healing effects to apply to all Rwandan civilians. This led to the decision that the intervention should be open to anyone who wanted to participate. This did not only impact the composition of sociotherapy groups, which as a consequence would not exclusively include mental health problem cases as defined through professional standards (like in Amsterdam). It also influenced the design of the effect study in a way which will be described in paragraph 2.

1.3 Instruments, study groups, measurements, informed consent

The study will follow a prospective non-randomized controlled concurrent groups design.
Data will be collected through structured interviews, with use of a questionnaire comprising various measurement instruments. Some of these are well-known and previously used instruments with proven validity. These have been, or will be, translated to the local language and validated for the context of the study. Other instruments have been, or will be, adapted or designed for the purpose of this study on the basis of local information, and will be validated as well.
The direct effects of the intervention will be established through interviewing its beneficiaries: the participants of sociotherapy groups. Secondly, as a proxy measure to the broader impact of the intervention, individuals in the social surroundings of group participants will also be interviewed. Finally, persons living in an area where no sociotherapy groups are meeting, will be interviewed to serve as controls. This results in the existence of three study groups:

1) DE: participants of sociotherapy groups, directly exposed (DE) to the intervention;
2) IE: individuals living close to the group participants, indirectly exposed (IE) to the intervention;
3) NE: individuals that are not reached by the intervention program: the control group, or non-exposed (NE).

The IE (indirectly exposed) group serves to test if the effect of sociotherapy on its participants also impacts individuals living close to these participants. This may then be considered as a proxy measure to the program's broader impact. Given the negative functional and behavioural consequences that trauma-related psychological problems generally have, a possible impact of the intervention on those who are closely related to its beneficiaries is worth studying. This obviously is the same for partner violence and alcohol abuse.

All three groups will be measured simultaneously at three subsequent moments, by use of the same questionnaire. As a baseline measurement the questionnaire will be taken at the start of the sociotherapy program (T0). Three months later (T1), directly after the sociotherapy groups have stopped meeting, all respondents will be taken an exit interview. As a follow-up, a third interview will be taken 6 to 8 months after the stop of the program (T2). In summary:

T0 = start of intervention
T1 = end of intervention (3 months after T0)
T2 = follow-up (6-8 months after T1).

At the start of each interview informed consent will be obtained by use of an explanatory text. Because of the high illiteracy in the study population this text will be read aloud, after which participation will be asked for. In case of refusal, some demographic data and reasons for refusal will be asked for and documented.

For our follow-up assessments questions will be added to collect data about other mental health or social services received.

1.4 Pilot study

The non-familiarity of the study's context to the researchers necessitated the collection of qualitative information, to help design a feasible methodology, and to help adapt or design measuring instruments which are adequate to the local semantics and culture. Therefore, this study has been preceded by a pilot study which took place from November 2005 to July 2007. Three periods of research activities in Byumba province, Rwanda, alternated periods of analysis in Amsterdam. A translator / assistent study coordinator was recruited locally. Through local focus group discussions and in-depth interviews information was obtained about the composition and distribution of the Byumba community; the existence and meaning of words and concepts; the comprehensibility and acceptability of questions; and social norms, values, manners and habits. Measuring instruments, adapted or designed with the help of this information, were translated, backtranslated, piloted and adapted again; external and internal validity was established.

Interviewers were recruited; four men and four women have been trained as interviewers for the pilot study. These are all Sociology students of the University of Byumba. During a one-week training period they were taught the principles of a longitudinal study design and made familiar with the measuring instruments and interviewing techniques. All were present at both measurement moments. A baseline and a follow-up measurement were carried out in three study groups (see above; DE:n=77, IE:n=63 and NE:n=90) to test the feasibility of sampling and interviewing methods. Collected data were analyzed and interpreted. Hard-to-interprete outcomes were discussed in another round of focus group meetings with community members, which once again led to instrument adaptations.

1.5 Intervention method

The intervention is a therapeutic group approach, tailored to survivors of systematic violence. Verbal exchange, debate, exercises, games and practical support are key elements. Trauma symptoms are addressed through support, psychoeducation and advise. Main goals are the restoration of safety, mutual respect, trust, care, and the setting of democratic rules. Groups contain 10 participants. Meetings are weekly over a period of 3 months, and last 3 hours each. Group leaders are local people, familiar with the region's history and current living situation; they have received a 3 months' training from Equator staff and are regularly supervised.
Group leaders have been trained during a 3 months period. There is daily program monitoring of, and support by the local supervisor, a pastor experienced in counselling and group work, who has great authority locally. He has an ongoing availability for consultation by group leaders in case of problems. He meets monthly with the 'leading team' of group leaders that has been installed. There is (minimally) weekly contact between him and the Dutch trainer/ supervisor, during which the program progress and possible problems are discussed. Group attendance by participants is well documented. The Dutch trainer/supervisor visits the program every 6 months, and provides a refresher training each year. She composed the exam that all group leaders will have to pass in August 2008 at the University of Byumba, to become officially certified sociotherapy group leaders.
Should any severe psychopathology become manifest in the course of a sociotherapy session series, the local program supervisor will be asked for assistance; reference to the local hospital, which has a psychiatric ward, will be considered. Sociotherapy group participants live in such small entities that a serious reason for no-show would be known among group members; also, no-show would be an immediate discussion issue within the group and be addressed through an outreaching support offer.

1.6 Interviews

The eight interviewers trained during our pilot study will be available for all three assessment periods. We will start with a training period as a refresher and to address the use of new(ly adapted) instruments. At the start of subsequent assessment periods a refresher training will be held also. The quality of interviewing will be assured by checking each questionnaire right after the interview, to see if there are any missing data. After each day of interviewing a meeting is held with all interviewers to discuss experiences and progress. Data are entered on the same day, so any questions arising while entering the data can be addressed the following day with the interviewer concerned.

2. DESIGN

2.1 STUDY POPULATION

2.1.1 Introduction

The study will be carried out in Byumba province in Rwanda, where 45 sociotherapy groups, each containing 10 participants, simultaneously start meeting weekly. After three months the intervention stops for these groups, and new series start for another 45 groups. All group participants are eligible for inclusion in the study, without any selection.

2.1.2 Inclusion criteria for sociotherapy groups

Within a 6 years period all areas of Byumba province will be covered by the sociotherapy program. The sequence is dictated by matters of actual convenience, and determined by the program's local counterpart. 'Areas' in Rwanda are mostly defined by natural boundaries (hills, trees) and historically determined social cohesion. There is no difference among areas in the amount of violence experienced.
Group participants are aged 16 years or older. The composition of groups is mixed (both sexes, various ethnic backgrounds, wide age distribution). No strict criteria for participation in a sociotherapy group exist. There are two routes towards participation: 1/ Sociotherapy group leaders, who are selected from persons generally regarded and accepted as leaders within their community, invite community members to participate on account of the seriousness of their psychosocial problems; 2/ Community members themselves apply for participation because they think it will help solve their psychosocial problems, and group leaders subsequently include them.
Apart from emotional problems arising from the past confrontation with atrocities and losses during thewar and genocide, community members currently experience a variety of problems such as poverty, bad housing, infectious diseases like HIV/AIDS, single parenthood, having to care for another family's children, having a spouse in detention, sudden arrests, bearing a curse from ancestors, neighbours or the family in law, etc. We have extensively discussed with group leaders what exactly would be the criteria to consider a person a problem case. It appeared to be extremely problematic for them to come up with such definition, the main reason being that it would automatically mean that people could also be excluded form group participation – a consequence they considered as undesirable. Another reason was formed by the still paranoid societal atmosphere in the region. People appear not to be willing to inform others about the nature of their problems or past traumatic experiences. They will only do so within the context of a sociotherapy group, after mutual confidence has been gained over a certain time. Therefore, it would be useless to ask detailed questions to possible future participants - it might even discourage participation.
As a result, no criteria to enter a group have been defined, other than being considered or acknowledged a serious problem case by group leaders. Consequently, no case definition exists on the basis of which a control group can be composed. This problem will be solved in the way described below (paragraph 2.2.3).

2.2 STUDY SAMPLE SELECTION

2.2.1 DE study group (n=100)

Out of 45 sociotherapy groups simultaneously starting the three months program, minimally 10 will be selected in a way that they represent rural as well as urban areas, are equally spread over the region, and in total contain an equal number of men and women. (Areas are called urban when they are part of Byumba town, but could also be considered rural as there is only one tarmac road in Byumba and people live on land in mud houses mostly. All areas are culturally similar. All were severely affected by the 1994 genocide.) All participants (n=100) of these 10 groups will be included in the DE group, without any further selection.

2.2.2 IE study group (n=100)

The IE study group (n=100) will be formed according to the following stepped procedure: 1) All respondents of the DE study group will be asked to list 5 people they live or work with and feel close to. These may be relatives they share the house with, neighbours they are daily in touch with, close friends they meet at least twice a week, or colleagues they closely collaborate with. 2)Then the collected lists will be blindly piled in a random order. 3)Then 1 person is selected from each list, by choosing the name of the 1st person from the first list, the 2nd from the second list, the 3rd from the third list, etc. After five subsequent lists, the 1st name of the sixth list will be selected, the 2nd of the seventh list, etc. Finally, the IE study group contains a number of people equal to the DE study group.

2.2.3 NE study group (n=100)

The NE study group (control group) will be randomly selected in a region where no sociotherapy groups are, or have been, meeting, or will be meeting over the coming 9 months. Non-intervention (control) areas are found at more than 10 miles away from intervention areas. As only a small number of people has the means to travel, there is no interaction on a regular basis between individuals in control areas and those in intervention areas.
The following convenience sampling method will be used: A similar proportion of urban and rural areas as in the experimental (DE) group will be selected. In all areas, interviewers will start at the top of a hill and each will walk down a different footpath towards scattered homes. They will randomly ask persons at home or in their fields to participate. They will interview an equal number of men and women per day. As no clear operational case definition can be formulated (see above), the matching of the experimental (DE) and the control (NE) group on 'caseness' wil be done on the basis of another criterion: an equal proportion in both groups of individuals scoring above cut-off on the SRQ-20 (the study's measuring instrument for probable psychiatric disturbance; see under 'Outcome Measures').
Data from the pilot study show that the prevalence of probable psychiatric cases in the DE and IE groups, as indicated through the SRQ-20, is 2.5 times higher than in the NE (control) group. On the basis of the assumption that this will not be different in the actual study, 2.5 times more respondents (n=250) will be interviewed for the NE (control) group than for the DE and IE groups.
After the first measurement and subsequent analysis of the data collected, we will select a subgroup of these controls with a similar mean SRQ score and sd at baseline as the intervention (DE) group, in order to establish comparable groups. This is possible due to the oversampling of the control group (n=250) for the baseline measurement. Also, this subgroup will be composed in such a way that it contains an equal amount of men and women with the same age distribution as the intervention group. If the intervention group does not have an equal sex distribution, we will look for a selection of controls with a similar distribution.
For the analysis of the IE group, we will select a subgroup of NEs with features that match the IE group in an exactly similar way as has been decribed above. This subgroup will then serve as the control group for the IE group.

2.3 OUTCOME MEASURES

During our pilot study we compiled a questionnaire consisting of three measuring instruments (see below). Through focus group discussions with local community members, each item was addressed with regards to relevance, comprehensibility and acceptability. Items were then adapted or added according to comments during these sessions. Finally the complete questionnaire was translated, back-translated, adapted again and piloted. In the upcoming study two instruments will be added to the pilot version of the questionnaire, to assess alcohol abuse and domestic violence. Primary outcome measure is mental health. Secondary outcome measures are social functioning, social capital, alcohol abuse and domestic violence.

2.3.1 Self Reporting Questionnaire 20 items (SRQ-20)

Primary outcome measure is mental health, as measured by use of the Self Reporting Questionnaire 20 items (SRQ-20). This is a structured interview developed by the World Health Organization (WHO) as a screening tool for common mental disorders in primary health care settings, especially in developing countries (ref.20,21). When patients are literate it can be self-administered, but in developing countries it is usually administered by lay interviewers. The instrument consists of 20 yes/no questions about mood, thinking capacity, feelings of anxiety and physical well-being. The SRQ-20 has been used in numerous settings (ref.20), also as a screening instrument in community samples. Cut-off points vary considerably depending on setting (community, primary care, hospital) and culture. A cut-off point of 8 is widely used (ref.22).

During our pilot study we validated the instrument locally (ref.23). We interviewed 99 respondents, who were also clinically assessed by clinicians who were blind for the SRQ-scores. We established a local cut-off score for caseness/non-caseness. The capacity of the SRQ-20 to identify probable psychopathology in this setting proved to be sufficient. The AUC was 0.76. When analysed separately for men and women the SRQ-20 showed to perform equally well in men (AUC=0.74) and women (AUC=0.76). In evaluating the instrument as a potential screener for psychiatric disorder the most appropriate cut-off point is a trade-off between a high sensitivity and an acceptable specificity. From our analyses the optimal cut-off point for the SRQ-20 appeared to be 10/11 (sensitivity 0.69; specificity 0.79). However, when men and women were analysed separately the cut-off points differed. The optimal cut-off point for men is 8/9 (sensitivity 0.69; specificity 0.65), while the optimal cut-off point for women is 10/11 (sensitivity 0.81; specificity 0.80). Reliability is considered to be good: alpha=0.83 (men: alpha=0.81; women: alpha=0.85).

2.3.2 Byumba Social Functioning Questionnaire (BSFQ)

One of the secondary outcome measures is social functioning. None of the standard instruments used world-wide to assess function ((MOS SF-36 (ref.24-26); WSDS (ref.27); DISS (ref.28); BDQ (ref.29)) fulfills suitability criteria for cross-cultural work. Those developed in western countries contain too many culturebound questions, eliciting responses that can be difficult to interpret across populations with varying physical requirements. Questions referring to ‘component’ activities such as lifting or walking may not directly assess the ability to complete tasks important to daily existence in a non-western context. Nor do any of the instruments acknowledge possible differences in the roles of men and women in low income countries.
In this study, the Byumba Social Functioning Questionnaire (BSFQ) will be used to measure social functioning. This instrument contains questions which have been derived from a list that resulted from previous research in Rwanda (ref.30). The actual questions have been compiled and listed during focus group interviews with people living in Byumba. Questions concern common daily activities for women and men, respectively, such as dressing oneself or taking care of the children. Data from our pilot study revealed that the instrument’s reliability is sufficient to good: alpha=0.77 (men: alpha=0.77; women: alpha=0.73).

2.3.3 MOS Social Functioning Scale 36 items (SF-36)

Social functioning will also be measured with the Medical Outcomes Study (MOS) Social Functioning Scale 36 items (SF-36) (ref.24-26). This internationally widely used instrument is based on the WHO definition of health. Within this definition, health is divided into three domains: physical, mental, and social health. The SF-36 contains questions about functional impairment related to physical or psychological problems. In this study the instrument will be used to further found the validity of the BSFQ. For use in Rwanda, items of the SF-36 will be adapted if containing contextually inadequate phrases (‘pushing a vacuum cleaner’, ‘walking blocks’, ‘playing golf’). Alternatives will be determined during discussions with local community members.

2.3.4 Social Capital Assessment Tool, short adapted version (SA-SCAT)

Another secondary measure is social capital, as measured by use of the Social Capital Assessment Tool, short adapted version (SA-SCAT). This instrument contains questions used to get an impression of the respondents’ connectedness to their social environment. They have been derived from a list of questions that resulted from previous research in various countries (ref.18) The actual questions were compiled and listed during focus group discussions with people living in Byumba. This has resulted in a locally adapted version. Questions concern the way people feel connected to others in their living area, feel supported by others, get along and undertake common actions with others. Data from our pilot study revealed that the locally adapted version consists of five factors: Active Membership, Cognitive Social Capital, Individual Support, Group Support, and Participation. These factors are congruent with previous research and theory (ref.18,31,32). Internal consistency of the factors ranges from 0.59 (Cognitive Social Capital) to 0.95 (Active Membership). In an effort to raise the instrument’s psychometric qualities, a version will be used during this study with items

added to its pilot study version, and yes/no answering options changed to Likert scales. Items to add will be determined during another round of discussions with local community members.

2.3.5 Alcohol Use Disorders Identification Test Consumption (AUDIT-C)

A third secondary outcome measure is alcohol abuse, as measured by use of the Alcohol Use Disorders Identification Test Consumption (AUDIT-C). This instrument contains 3 questions assessing the number and frequency of having 'drinks containing alcohol', a formulation which is suitable for use in a country where alcohol may often not be consumed in identifyable units like commercial bottles or standard glasses. The AUDIT-C has shown to be an effective screening instrument for alcohol misuse on primary care level (ref.33). Subsequent validation in American clinical outpatients (ref.34,35) and in European samples (ref.36-38), respectively, yielded different optimal screening thresholds. The instrument has not been validated locally during the pilot phase of this study. Therefore, after translation and back-translation, the instrument will only be used to explore change in alcohol use over time.

2.3.6 Revised Conflict Tactics Scales, short form (CTS2S)

A final secondary outcome measure is partner violence, as measured by using elements of the Revised Conflict Tactics Scales, short form (CTS2S, ref.39): the 'negotiation', 'psychological aggression', 'physical assault' and 'injury' scales. (The remaining scale concerns sexual coercion, a subject inacceptable to address in a short, one-time interview in Rwanda.) The CTS2 is the most widely used and men, respectively, such as dressing oneself or taking care of the children. Data from our pilot study revealed that the instrument's reliability is sufficient to good: alpha=0.77 (men: alpha=0.77; women: alpha=0.73). The CTS2 is the most widely used instrument for measuring family violence (ref.40,41). The short form enables the CTS2 to be used when testing time is limited. There is only a clear indication so far that the validity of the CTS2S is comparable to the CTS2, and the CTS2S has not been validated locally during the pilot phase of this study. Therefore, the instrument will be considered to only provide an indication of partner violence. The instrument classifies respondents into three severity levels: 'none', 'minor only' and 'severe', and creates mutuality types: 'male partner only', 'female partner only', and 'both aggressive'. The latter is relevant as in this study only one of the partners of possible couples will be interviewed. As the 7-options-per-item answering system of the CTS2S is considered too complicated for the study population to provide valid answers, an adaptation to this will be designed and piloted after translation and back-translation.

3. SAMPLE SIZE AND POWER

The primary hypothesis to be tested is that there is a significant increase in mean SRQ score of the intervention group as compared to the control group. 100 respondents per treatment condition will be included in the study, 300 respondents in total. This sample size calculation is derived from data from our pilot study. The pilot study showed an increase of 2.7 (sd 4.2) in mean SRQ score in the intervention (DE) group, which is considered clinically significant (effect size 0.6). To establish a 2.7 effect with a standard .80 power, a minimum of 30 respondents per condition is needed. For several reasons we will aim at larger numbers of respondents (n=100) per study group: *to be on the safe side in case of drop-out, problems with interviewing and scoring, missing data, etc.; *to compensate for psychometric scale properties that are not optimal; *to be able to stratify, e.g. into male and female subgroups.

4. ANALYSIS

Preliminary analysis will be performed to check the (normality of the) distribution of the variables and check for systematic drop-out between the measurements. A three by two MANOVA analysis will be used to test change over three periods of time. Multivariate analyses will be used to adjust for potentially correlated variables (i.e. respondent characteristics). Relationships between the variables will be analysed using Pearson correlation coefficients.

Expertise, prior activities and products

The study will be carried out within the framework of the Topzorgprogramma (TZP) Psychotrauma at AMC de Meren, which the Equator treatment program belongs to. The TZP is a program for scientific research and patient care in the field of psychiatric disorders related to traumatic events. Its scientific program is part of the AMC research program 'Chronicity and Care Programs in Psychiatry'.
D. Denys, who will act as the promotor, is an expert in the field of anxiety disorders. He is (co-) author of over 45 international scientific publications. With his thesis 'On Certainty; studies in obsessive compulsive disorder' he acquired his PhD degree with a 'cum laude' annotation. In 2005 he received the Ramaer decoration of the Dutch Psychiatric Association NVvP for his excellent and innovative research. From 2004 to 2006 he was head of the Anxiety & Compulsive Disorders program of the department of Psychiatry at the University Medical Center in Utrecht. Since March 2007 he is the head of the department of Psychiatry at the AMC in Amsterdam.
W. Scholte, who will act as the project leader, is (co-)author of 14 international scientific publications. He has broad expertise in international mental health interventions, and will shortly finish a PhD thesis on related studies. For 12 years he was the initiator and advisor of mental health programs in (post-)war situations for Doctors Without Borders. With others he published an evaluation and an epidemiological mental health survey concerning Rwandan refugees living in camps in Tanzania. For War Child Netherlands he acted as a content advisor and program evaluator. In 2004 he and others performed and published an epidemiological mental health survey in Afghanistan. Scholte participates in an international research group performing a longitudinal study on stress in humanitarian aid workers. He is one of the founders of the Transcultural Psychiatry platform of the NVvP. In 2003 he started off and became the head of Equator, the AMC treatment program for traumatized refugees. In 2003 the treatment program was granted financial funding by the European Refugee Fund. In 2004 the Centers for Disease Control and Prevention (CDC) in the USA granted funding for an epidemiological study in Afghanistan. In 2005 funding was gained from Cordaid (development organization) to execute the intervention program in Rwanda. In 2006 a 3 years longitudinal effect study on the Equator treatment program at the AMC was granted funding by the European Refugee Fund. Scholte has a broad network of (inter-)national mental health experts, ranging from colleagues operational in Dutch refugee mental health care and in care programs in development countries to researchers and policy advisors e.g. at WHO, Institute of Psychiatry in London, London School of Hygiene and Tropical Medicine, Columbia University in New York and the University of Melbourne.
A. Richters is a physician and medical anthropologist. She works as professor in culture, health and illness at the Leiden University Medical Center, Department of Public health and Primary care, Discipline group Medical Anthropology. The subject of her previous professorship at the same Center was 'women and health care'. She finished studies in subsequently medicine, anthropology, sociology and philosophy (the last three cum laude), culminating in 1991 in an interdisciplinary PhD thesis "The medical anthropologist as narrator and translator" (written in Dutch) (cum laude). After having worked for a number of years as a physician in various positions in the Netherlands and abroad, she held various positions in different departments (philosophy and medical ethics, applied sociology, cultural anthropology, women and autonomy) at subsequent different universities in the Netherlands. At present she supervises a number of PhD students with topics ranging from the role of culture in mental health care, the experience of refugee women in the Netherlands with sexual violence during war, women suffering from infertility in Bangladesh, and community recovery strategies after war in Mozambique, to displaced children's illness experiences and quest for therapy in northern Uganda. Themes in her present teaching and research are: gender violence, trauma, health and healing; war, health and human rights; medicine and human rights in cross-cultural perspective; intercultural medicine, and the quality of reproductive health care for migrant women in the Netherlands.
M. Olff is associate professor in the field of psychological trauma. She is head of the TZP Psychotrauma at AMC de Meren. She has a visiting professorship in Norway. She is (co-)author of 40 international scientific publications. She is on the board of the European Society for Traumatic Stress Studies (ESTSS). Her research projects include randomized controlled trials on different forms of debriefing, early intervention and psychotherapy in traumatized patients; psychobiological research incl. neuroimaging and neuroendocrine studies; disaster related research; an epidemiological survey on trauma and PTSD in the Netherlands; a study on posttrauma psychopathology at the AMC surgical

trauma unit; a European project on post disaster psychosocial care.
A. Kamperman is an expert on migrant and refugee mental health research. Her research experience is focused on the development of sampling strategies for research populations who are difficult to reach and the adaptation of instruments for use in non-western and ethnic diverse populations. Her PhD research was a large scale epidemiological health survey among the migrant population of Amsterdam. The research's objective was to assess general and migrant specific determinants of mental health and mental health care consumption. She has much experience with development and coordination of projects aimed at psychological wellbeing and health within in these populations.
Th. Rutayisire is a Rwandan psychologist who did his masters in South-Africa. He is fluent in English. He is a lecturer at the Institut Polytechnique de Byumba. He acts as the local counterpart in the coordination and as the translator for the Dutch researchers of the Equator study in Rwanda.
F. Verduin, candidate researcher, has shown consistent interest in international mental health over time; she was a junior researcher and co-author of a related scientific publication during her medical training; as a resident in psychiatry she helped shape the Equator treatment program for refugees in its starting phase; to the pilot of the present study she contributed from the start with relevant technical and practical input.

References

1. Strang A & Ager A (2003). Psychosocial interventions: some key issues facing practitioners. Intervention 3:2-12.
2. Mooren T, de Jong K, Kleber R, Ruvic J (2003). The efficacy of a mental health program in Bosnia-Herzegovina: impact on coping and general health. J Clin Psychol 59:57-69.
3. Bolton P, Bass J, Betancourt T, et al (2007). Interventions for Depression Symptoms Among Adolescent Survivors of War and Displacement in Northern Uganda: A Randomized Controlled Trial. JAMA 298: 519-527.
4. Bass J, Neugebauer R, Clougherty KF, et al (2006). Group interpersonal psychotherapy for depression in rural Uganda: 6-month outcomes. Br J Psy 188:567-573.
5. Neuner F, Schauer M, Klaschik C, et al (2004). A comparison of narrative exposure therapy, supportive counseling, and psychoeducation for treating posttraumatic stress disorder in an African refugee settlement. J Consult Clin Psychol 72:579–587.
6. Miller K, Kulkarni M, Kushner H (2006). Beyond trauma-focused psychiatric epidemiology: bridging research and practice with war-affected populations. Am J Orthopsychiatry 76:409-422.
7. Scholte WF, Olff M, Ventevogel P, et al (2004). Mental health symptoms following war and repression in eastern Afghanistan. JAMA 292:585-93.
8. Silove D (1999). The psychosocial effects of torture, mass human rights violations, and refugee trauma: toward an integrated conceptual framework. J Nerv Ment Dis 187:200-207.
9. Ager A (2002). Psychosocial needs in complex emergencies. Lancet 360:s43-44.
10. Bolton P, Neugebauer R, Ndogoni L (2002). Prevalence of depression in rural Rwanda based on symptom and functional criteria. J Nerv Ment Dis 190:631-637.
11. Schaal S, Elbert T (2006). Ten years after the genocide: trauma confrontation and posttraumatic stress in Rwandan adolescents. J Trauma Stress 19:95-105.
12. Pham PN, Weinstein HM, Longman T (2004). Trauma and PTSD Symptoms in Rwanda: Implications for Attitudes Toward Justice and Reconciliation. JAMA 292: 602-612.
13. Hagengimana A, Hinton D, Bird B, et al (2003). Somatic panic-attack equivalents in a community sample of Rwandan widows who survived the 1994 genocide. Psychiatry Res 117:1-9.
14. Dyregrov A, Gupta L, Gjestad R, Mukanoheli E (2000). Trauma exposure and psychological reactions to genocide among Rwandan children. J Trauma Stress 13:3-21.
15. de Jong JP, Scholte WF, Koeter MW, Hart AA (2000). The prevalence of mental health problems in Rwandan and Burundese refugee camps. Acta Psychiatr Scand 102:171-177.
16. http://www1.worldbank.org/prem/poverty/scapital/index.htm
17. Putnam R (1995). Bowling Alone. Journal of Democracy 6.1: 65-78.
18. Harpham T, Grant E, Thomas E (2002). Measuring soial capital within health surveys: key issues. Health Policy and Planning 17:106-111.
19. Bolton P, Stichick Betancourt P (2004). Mental health in postwar Afghanistan. JAMA 292:626-628.

20. Beusenberg M & Orley J (1994). A user's guide to the Self Reporting Questionnaire (SRQ). Geneva: World Health Organization, Division of Mental Health.
21. Harding TW, Arango MV, Baltazar J, et al. (1980). Mental disorders in primary health care: a study of the frequency and diagnosis in four developing countries. Psychol Med 10:231–242.
22. Harpham T, Reichenheim M, Oser R, et al. (2003). Measuring mental health in a cost-effective manner. Health Policy Plan 18:344–349.
23. Scholte WF, Kamperman AM, Verduin F et al. Longitudinal validation of the Self Reporting Questionnaire 20 items (SRQ-20) in a Rwandan community sample. Submitted.
24. Ware JE Jr (1993). SF-36 health survey manual and interpretation guide. The Health Institute, New England Medical Center, Boston,MA.
25. Ware JE Jr, Kosinski M, Bayliss MS, et al (1995). Comparison ofmethods for the scoring and statistical analysis of SF-36 health profile and summary measures: summary of results from the Medical Outcomes Study. Medical Care 33: AS264–AS279.
26. McHorney CA,Ware JE Jr (1995). Construction and validation of an alternate form general mental health scale for the Medical Outcomes Study Short-Form 36-Item Health Survey. Medical Care 33: 15–28.
27. Mauskopf JA, Simeon GP, Miles MA, et al (1996). Functional status in depressed patients: the relationship to disease severity and disease resolution. J Clin Psychiatry 57: 588–592.
28. Sheehan DV, Harnett-Sheehan K, Raj BA (1996). The measurement of disability. Int Clin Psychopharmacol 11 (Suppl 3): 89–95.
29. Ormel J, VonKorff M, Oldehinkel AJ, et al (1999). Onset of disability in depressed and non-depressed primary care patients. Psychol Med 29: 847–853.
30. Bolton P & Tang M (2002). An alternative approach to cross-cultural function assessment. Soc Psychiatry Psychiatr Epidemiol 37:537-543.
31. Harpham T, Wilkinson R, Mckenzie K (2006). Social Capital and Mental Health. London: Jessica Kingsley Publishers.
32. De Silva MJ, Huttlya SR, Harpham T, et al (2007). Social capital and mental health: A comparative analysis of four low income countries. Soc Sci Med 64: 5–20.
33. Bradley KA, DeBenedetti AF, Volk RJ, Williams EC, Frank D, Kivlahan DR (2007). AUDIT-C as a brief screen for alcohol misuse in primary care. Alcohol Clin Exp Res 31: 1208-1217.
34. Bradley KA, Bush K, Davis TD, et al (2003). Two brief alcohol screening test from the Alcohol Use Disorders Identification Test (AUDIT): validation in a female VA primary care patients. Arch Intern Med 163: 821-839.
35. Bush KR, Kivlahan DR, McDonell MB, et al (1998). The AUDIT alcohol consumption questions (AUDIT-C): an effective brief screening test for problem drinking. Arch Intern Med 158: 1789-1795.
36. Aertgeerts B, Buntinx F, Ansoms S, et al (2001). Screening properties of questionnaires and laboaratory tests for the detection of alcohol abuse or depence in a general practice population. Br J Gen Pract 51: 206-217.
37. Gual A, Segura L, Contel M, et al (2002). Audit-3 and audit-4: effectiveness of two short forms of the alcohol use disorders identification test. Alcohol Alcohol 37: 591-596.
38. Rumpf HJ, Hapke U, Meyer C, et al (2002). Screening for alcohol use disorders and at-risk drinking in the general population: psychometric performance of three questionnaires. Alcohol Alcohol 37: 261-268.
39. Strauss MA, Douglas, EM (2004). A short form of the Revised Conflict Tactics Scales, and typologies for severity and mutuality. Violence Vict. 19: 507-520.
40. Strauss MA, Hamby SL, Boney-McCoy S, et al (1996). The Revised Conflict Tactics Scales (CTS2). J Fam Issues 17: 283-316.
41. Straus MA (2004). Bibliography and tabular summary of publications on the Revised Conflict Tactics Scales (CTS2 and STSPC). Durnham, NH: Family Rersearch Laboratory, Univ of New Hampshire.
